# "The Hospital" Medical Book Supplement.—No XXV

**Published:** 1910-01-15

**Authors:** 


### "THE HOSPITAL"
### MEDICAL BOOK SUPPLEMENT.—No XXV.

CONTENTS.

PAGE

**Medicine—**

Remedial Gymnastics for Heart Affections, used at Bad-nauheim ... ... ... ... ... ... ... ... ... ... 453

Pnlmonary Tuberculosis and Sanatorium Treatment ... ... 453

Soured Milk and Pure Cultures of Lactic Acid Bacilli in the Treatment of Disease ... ... ... ... ... ... ... 453

A Handbook of Medical Diagnosis: for the Use of Practitioners and Students ... ... ... ... ... ... ... ... 454

Diseases of the Stomach ... ... ... ... ... ... ... ... 454

PAGE

**Surgery—**

A System of Operative Surgery ... ... ... ... ... 455

Urgent Surgery ... ... ... ... ... ... ... ... 455

**Miscellaneous Items—**

Health in the Home ... ... ... ... ... ... ... 456

The Periodic Law ... ... ... ... ... ... ... ... 456

**Reference—**

The Midwife's Dictionary ... ... ... ... ... ... ... 456

Primer of Statistics ... ... ... ... ... ... ... 456

### MEDICINE.

REMEDIAL GYMNASTICS FOR HEART AFFECTIONS, USED AT BAD-NAUHEIM. By Dr. HOFMANN and Dr. PÖHLMAN. Translated and Edited by Dr. J. G. GARSON. (London: Swan, Sonnenschein and Co., Ltd., 1909. Price 5s. net.)

THIS book is a translation of Hofmann and Pöhlman's work entitled "Die Gymnastik der Herzleidenden." We have recently reviewed a very similar volume upon the same subject. The letter-press of the book before us occupies but forty pages, and the rest consists of full-page illustrations of the various passive and active exercises that are recommended. One of the main points insisted upon by the author is that quite as much harm as good may be done by the Nauheim treatment unless very particular care is taken first of all in selecting the cases to be so treated; secondly, in carefully avoiding the tendency there is to do too much and to advance from stage to stage too rapidly; and thirdly, unless the effects of the exercises are checked by careful supervision of the pulse, blood pressure, physical signs, *x*-ray appearances of the heart, and so on. The author devotes his fifth chapter to an account of an ingenious apparatus, invented by Sommer, for recording the involuntary tremble of the legs in different planes when the patient sits down in a chair after the exercises; these tremblings show different characters according to the degree of fatigue reached, and the records of them would seem to afford valuable means of checking the effects of the treatment. As the author points out, however, this is an investigation which has not yet been completed, although the results obtained so far seem to indicate that it is valuable. The last chapter of the book is devoted to a discussion of the order in which the exercises should be practised, special exercises, the importance of breathing properly during the exercises, and the question of diet and stimulants.

PULMONARY TUBERCULOSIS AND SANATORIUM TREATMENT. By C. MUTHU, M.D., M.R.C.S., L.R.C.P. (London: Baillière, Tindall, and Cox. 1910. Price 3s. 6d. net.)

THERE are a good many publications upon the subject, the main features of most of them being very similar; nevertheless the personality of different authors is bound to differ, so that the subject is presented by each of them in a somewhat different light. We think Dr. Muthu's book will prove distinctly serviceable even to those who possess other recent works upon the same subject. The volume is in three parts. Part I. deals with the predisposing factors of tuberculosis, the communicability of pulmonary tuberculosis, its early diagnosis and prognosis. Part II. deals with the principles of open-air treatment, the results of sanatorium treatment, the requirements of an ideal sanatorium, the treatment of hæmoptysis, the continued inhalation treatment of laryngeal tuberculosis, and the formaldehyde and static electric treatment of consumptives. Part III. deals with the social and economic factors of tuberculosis, the remedial and preventive measures, from a public point of view, and the relation of marriage to consumption. The author deprecates the present tendency of the public to look askance at any tuberculous subject, and he lays stress upon the fact that practically every individual contains tubercle bacilli, though only a few suffer from the ravages of the infection; the ultimate factor in the development of phthisis is the resistance of the individual, and upon this fact Dr. Muthu insists all through his book. The result is that he is lenient in his views as to the marriage of consumptives, for example, and as to the liability of the offspring of phthisical persons to develop consumption. It is clear throughout the book that the interests of the patients themselves are invariably given the first place. There is a chapter devoted to arguments against the notification of phthisis, and we hope to comment upon this again later, and also perhaps to refer to Dr. Muthu's views upon marriage in relation to consumptives.

SOURED MILK AND PURE CULTURES OF LACTIC ACID BACILLI IN THE TREATMENT OF DISEASE. By GEORGE HERSCHELL, M.D. (London: H. J. Glaisher. Chicago: Chicago Medical Book Company. Second Edition. 1909. Price 2s. 6d. net.)

THE rapid exhaustion of the first edition of this book has given the author the opportunity to revise it thoroughly for the present edition. Although without doubt the form of treatment to which this publication directs attention is valuable for certain conditions, and does great good within well defined limitations, there is no disguising the fact that it has become the fad of the moment, more especially among the laity. Dr. Herschell has done well to lay down more precisely than before what those limitations are, and how it is to be determined whether a given patient is likely to be benefited by soured milk or not. He has done this with the object of stopping, if possible, the promiscuous adoption of commercial sour milk diet by the public at large for real or fancied ailments; but we cannot help suspecting that a good proportion of those who exhausted the first edition of Dr. Herschell's work are members of the general public, and not medical men. However, it gives us pleasure to testify to the moderation and sense with which he presents his case to the profession. He does not allow enthusiasm to run away with him, and in its present form the monograph is one which any doctor who thinks of trying this treatment will do well to study.

A Handbook of Medical Diagnosis: for the Use of Practitioners and Students. By J. C. Wilson, A.M., M.D. (Philadelphia and London: J. B. Lippincott Company. 1909. 25s. net.)

We have no great belief in the desirability of books on general diagnosis. The art of correct recognition of the varieties of disease can only be learned at the bedside, and the essentials of that art, however well they may be summarised and described in textbooks, can only be mastered under the personal supervision of the teacher. No amount of reading will enable a student to distinguish between the dulness caused by fluid and that due to solid lung, or between a moist and a crepitant râle. Nevertheless, there seems to be a popular professional taste for such handbooks, especially in America; and the authors in nearly every case have done their solid best to make their labours really instructive and helpful. As models may be cited the authoritative works of Sahli and Da Costa, both of which are doubtless well known to practitioners. Dr. Wilson's book is no exception to the general rule. It is the work of a careful, methodical, painstaking compiler. There is no great originality displayed in the style or the manner in which the instruction is conveyed, and the book therefore does not compare favourably in that respect with the two authors already cited. Its merit is that it is fairly up to date and that its teaching is everywhere sound. As is usual in such works, there is an immense amount of padding: half of the book could have been excised without interfering with its usefulness. The illustrations are well chosen, many of them distinctly original and elucidative of the text. In many sections the diagnosis is imperfectly discussed and treated in much too elementary a fashion. As an example may be quoted the chapter on pancreatic diseases, where no mention is made in detail of the various tests beyond a two-line statement about the glucose test and the assertion that Cammidge's reaction is much too complicated to be dealt with. On the other hand, the chapters dealing with the nervous system are full and instructive: they are perhaps the best the book contains. There is, of course, little said about treatment, while ætiology and pathology are very briefly dealt with. As a reference book the work has a value, and practitioners will find it useful when they are in doubt. We cannot assert, however, that it has a higher value or possesses greater merits than the more generally known handbooks on differential diagnosis.

Diseases of the Stomach. By S. H. Habershon, M.D. (London: Cassell and Co. 1909. Pp. 565.)

The textbook which Dr. Habershon now presents as a manual for practitioners and students, is his answer to numerous requests which have reached him to republish his father's works on "Diseases of the Abdomen." Probably this decision is a sound one, for to edit a textbook more than twenty years old would involve so much alteration, both in substance and arrangement, that the original would be quite unrecognisable. The result at any rate is a monograph devoted entirely to gastric disorders, or, rather, to their medical aspects, for naturally one does not look for details of operative surgery from a physician. Notwithstanding this, the omission of the surgical aspects of diseases of the stomach seems a mistake, in view of the success which has attended operative procedures in certain conditions. Although Dr. Habershon recognises fully and generously the results of modern gastric surgery when intelligently conducted, his outlook is too restricted. Here and there, for instance, he recommends gastro-enterostomy for various diseases; but nowhere is there discussion of the operation, the indications for and against it, the causes of non-success, the complications or sequelæ, or, in fact, of the results generally. Dr. Habershon's plan is, of course, that hallowed by custom, but the advantages of combining medicine and surgery in a regional textbook are very great, as the editors of the Medico-Chirurgical Series now appearing have recognised.

Within the limitations to which the author restricts himself his work is good. Preliminary chapters on the anatomy and physiology of digestion, on nutrition, and on diet in health and in disease are long, but not at all too long considering the importance of the subjects. The examination of the stomach and of the gastric contents, with the symptomatology of gastric disease, are then discussed. These chapters are excellent, and contain many hints of practical value. A curious method by which Dr. Habershon is accustomed to distinguish spurious from real gastric pain is to compress one of the carotid arteries in the neck, preferably the left; this is said to relieve the pain in functional but not in organic cases. In dealing with the estimation of hydrochloric acid in the stomach the author does not make clear the uselessness of any such investigation unless conducted by an expert. For practitioners or students to attempt these analyses is sheer waste of time, and the importance to be attached to the results, even when they can be relied on, is probably over-estimated by Dr. Habershon.

It is on the complex subject of dyspepsia that any writer on disorders of the stomach is most likely to offer a target for criticism. Bearing in mind the extreme difficulty of treating this intricate question adequately and yet concisely, we feel inclined to say that the author has tried very hard, but just not succeeded in triumphing over it. The eighty pages devoted to dyspepsias are full of careful work, yet their perusal renders one a little tired and a little puzzled. Probably a slightly more dogmatic teaching, if less conscientious, might have been more valuable to those for whom these pages are written. Treatment is treated a little scurvily, after the manner of many current textbooks; yet the successful treatment of dyspepsia is an asset in general practice second, perhaps, to no other qualification whatever. The teaching is quite sound throughout, only it is not quite explicit enough. In the remaining chapters on the many other diseases of the stomach the author is seen to more advantage; on gastric ulcer and gastric cancer his careful sections are excellent indeed. One of the very few instances in which we cannot quite follow him is concerned with the after-breakfast smoke and its bearing on defæcation. Tobacco-smoking, he says, produces relaxation of the muscular fibres of the stomach and intestines, and this is no doubt the cause of an action of the bowels after breakfast. Surely the emptying of the intestine demands active peristalsis not paresis of the bowel wall? The conception of gravity as the agent by which, when the intestines relax, the fæces are withdrawn seems to be novel and unorthodox. It is so rare to find a slip that one fancies the author's meaning may be something quite different. His interesting monograph is one which will, we feel sure, find a place in very many practitioner's libraries.

### SURGERY.

A System of Operative Surgery. Edited by F. F. Burghard, M.S. (Lond.), F.R.C.S. (Eng.); Teacher of Operative Surgery in King's College, London, Surgeon to King's College Hospital, and to the Children's Hospital, Paddington Green. In four volumes. Vol. III. (Henry Frowde and Hodder and Stoughton, London, 1909. Pp. xxxii. + 764. Illustrations, 381. Price, 36s.)

In this, the third volume of Mr. Burghard's system of Operative Surgery, the high standard of excellence which distinguished the two volumes previously published is actually surpassed. This is partly due to the fact that it deals more with those operations which come within the scope of a general surgeon, as opposed to those which are rarely performed, or are admittedly the province of specialists, as in volumes I. and IV. The reader who comes to this work for assistance is really helped, and is not left to make up his own mind as to the relative merits of the various procedures which are open to him; and this method is satisfactorily maintained without any suggestion of narrow-mindedness or dogmatic utterances. The volume opens with an article upon the surgical treatment of tuberculous disease of the lymphatic glands, by Mr. Harold Stiles. Comparisons are admittedly odious, but we are compelled to express our admiration for this article, which is the best in the book. It can hold its own with anything in recent medical literature. He gives an extremely clear description of the anatomy of the lymphatic glands of the neck, and he proceeds to point out that the results of removal of tuberculous glands in the neck, if properly performed, are exceedingly encouraging. The operation must be thorough, and if the glands are caseating, it is not necessarily sufficient to evacuate the caseous material and to trust to luck that the deeper portions of the disease will be removed by natural processes. The operations upon the thyroid gland (spelt throughout "thyreoid," which is not more correct, and is certainly pedantic) are described by Mr. Berry, admittedly one of the greatest authorities on the subject in this country. His article upon extirpation of the thyroid gland, and particularly his practical remarks upon its difficulties and dangers, should be read by every surgeon who may be called on to perform this operation. The diagrams illustrating it are particularly good. Mr. Mayo Robson is the author of the chapter dealing with the bile passages and the pancreas. That in itself is sufficient guarantee of its excellence. His remarks upon the value of draining the large peritoneal pouch, which lies on the right side of the abdomen, between the lower surface of the liver and the transverse mesocolon, are particularly interesting. The case for cholecystostomy against cholecystectomy is well stated, and his description of cholecystenterostomy by simple suture is unusually lucid. Mr. Rawling deals effectively with operations upon the skull and brain. He shows an admirable restraint in considering the merits and demerits of operations for the removal of cerebral tumours, and we are much impressed by the value of Cushing's "Intermusculo-temporo-cerebral decompression," as described by him. To illustrate the divergence of opinions which surgeons may possess, he humorously quotes three diametrically opposed views as to the value of the hand trephine. The operations upon the spinal cord and canal are adequately described by Mr. Thorburn, of Manchester; but an unnecessary amount of space is given to the methods of uniting the vertebræ with wire, an operation rarely performed, and more rarely called for. The chapter on the surgery of the kidneys comes from Dr. Newman, of Glasgow. It is particularly good on the methods of physical examination of the kidneys and ureters. There is a clear account of the technique of cystoscopy, with some useful coloured diagrams representing the various aspects of the ureteric orifices in renal disease. Mr. Thomson Walker is responsible for the diseases of the bladder. His account of the method of removal of vesical tumours, both malignant and non-malignant, is admirable. Mr. Freyer contributes an article upon vesical calculus. More space is given to lateral and median than to suprapubic lithotomy, which is curious in view of the fact that the latter operation has almost entirely superseded the other two. His chapter upon diseases of the prostate is, however, excellent. The editor supplies an account of the operations on the male genital organs. His description of the plastic treatment of the congenital malformations of the penis is a model of lucidity. We cannot, however, agree with his partiality for orchidopexy for retained testis. The late results of this operation are most unsatisfactory. It is quite the exception to meet with a case in which the testicle has remained and developed normally in the scrotum after this procedure. Mr. Stiles gives an excellent description of the radical operation now in vogue for carcinoma mammæ; and, finally, Mr. Godlee describes the surgical operations which can be performed on the thoracic viscera. On the whole, the third volume of this work is the most instructive of those yet published, and should form a most valuable asset to anyone engaged in the practice of surgery.

Urgent Surgery. By Professor Felix Legars. Translated from the Sixth French Edition by William S. Dickie, F.R.C.S. (Bristol: John Wright and Sons, Ltd. 1909. Vol. I. 25s. net.)

To surgeons all the world over Professor Legars' volume on emergency surgery is well known. It is a work that has been translated into all languages that possess a scientific literature, and the wonder has been that it has not before been translated into English. Perhaps the delay has been fortunate to the author in that it has brought him so excellent an interpreter as Mr. Dickie. For we must preface all remark by cordially congratulating translator and publisher on the excellence of their united accomplishment. This first volume of the English edition is a model of what such works should be. Admirably printed, more admirably illustrated, and, above all, written in a lucid, individual style which is not a mere literal Englishing of the terse French that the surgeon of St. Antoine wrote, these 600 odd pages are a delight to read. We do not exaggerate when we declare that it is a work that should be familiar to every surgeon, and that general practitioners, whose knowledge of French is insufficient to enable them to follow Legars in the original, should not hesitate to buy this translation for a permanency on their bookshelves. In the first volume the emergency surgery of the head, neck, thorax, spine, and abdomen is treated. The introductory chapter, dealing with asepsis and anæsthesia is especially well written. It must be borne in mind that this technique is that in vogue in the French schools, for Mr. Dickie has not gone out of his way to compare the author's methods with those that are practised in England. For that reason one used to the English routine—or for that matter to the German or Italian schools—may demur here or there, but the passages to which exception can be taken contain more what are matters of opinion than expressions of dogmatic teaching. Thus we may refer to the really helpful article on tracheotomy (which is essentially in the majority of cases an operation of urgency), in which it is stated that "when it is all important to avoid loss of blood . . . the operation may be performed with the thermo-cautery." The candidate who ventures to suggest that to an examiner in surgery at the conjoint examination will probably be mulcted in marks, but it is a method that is sometimes used in French hospitals, though it is scarcely one that we should venture to commend to the general practitioner. Short *résumés* of interesting illustrative cases from the author's own books and from the literature are given, and these certainly increase the practical value of the book. As specially interesting points, we may refer to the electric enema, which is so little used in this country; whereas in France it has been found of real service in the treatment of intestinal conditions. The chapters dealing with the surgery of the abdomen, though necessarily far from comprehensive, are very clearly written, and instructive from all aspects; the portion dealing with the diagnosis, prognosis, and treatment of appendicitis is particularly ably elaborated. Equally good are the chapters dealing with thoracic lesions; in these much material that will be comparatively novel to many readers has been made use of, more particularly in the sections relating to cardiac surgery and operations on the lungs. Here and there an explanatory note might have been of use to readers. How many English practitioners, for instance, know what a Bonnet's trough is, or are able to make or apply it? In the section dealing with laryngeal work, some description of laryngoscopy, œsophagoscopy, and bronchoscopy might reasonably have been expected. We cordially commend the work to every general practitioner, to every student, and, although it is primarily more adapted to the two former classes, to the operating surgeon. Surgical residents, for example, will find it an immensely useful and interesting book, and it should find a place in every college library. We look forward with pleasurable anticipation to the second volume, which is promised early in the present year.

### MISCELLANEOUS.

Health in the Home. By J. Johnston, M.D. (Manchester: John Heywood, Ltd. 1909. Price 6d. net.)

This attempt to instruct the public, especially the working classes, in the elementary laws of health and hygiene is one for which a wide circulation may be hoped. As a sane and sensible effort to combat the ignorance and superstition which account for much of the deplorable physical degeneracy of the people, published at a price which places it within the reach of everyone, Dr. Johnson's pamphlet deserves the fullest recognition. It is well arranged, comprehensive, and salutary; but it never trenches on the proper province of the doctor in the home treatment of sick children, and it very rightly contains more about prevention than about cure. The only objection we have to urge is that now and then the language is unnecessarily technical. Thus parturition is a word whose meaning many mothers, even in the educated classes, may not understand; and other scientific terms are used for which the equivalents in Anglo-Saxon might with advantage be substituted. In the account of milk mixtures for infants at various stages of life it would probably have been better to describe the quantities of milk, water, etc., necessary for a single feed rather than to give larger amounts and then to direct that so many ounces should constitute one feed.

The Periodic Law. By A. E. Garrett, B.Sc.Lond. The International Scientific Series. Edited by F. Legge. (Published by Kegan Paul, Trench, Trübner and Co. 289 pp. with index, tabular illustrations, etc.)

There seems to be a distinct tendency, as fresh numbers are added to this series, to become more technical, and to eschew the popular exposition which characterised the earlier works. Whether this is deliberate, or arises inevitably out of the subject, the present volume could scarcely appeal to one who had not already an extensive knowledge of chemistry. To those however who possess the necessary elementary knowledge, this book will be of considerable value. The Periodic Law is perhaps one of the most remarkable discoveries of modern science, and the author has traced lucidly, though briefly, the gradual steps by which these conclusions were reached. The historical account of the Periodic Law ends with the work of Carnelly, the rest of the book, *i.e.* Chapters VII.-IX., dealing with its application to modern research. Chapter IX. gives a most interesting discussion of the nature of the atom, in view of the recent work of Sir J. J. Thomson. This chapter might conceivably have been lengthened, for to the uninitiated the formulæ are too quickly introduced, and unless the reader has considerable experience in this kind of calculation, will be of little service. The diagrams and tables are, however, a praiseworthy feature and tend to lighten any obscurity of the text.

### REFERENCE.

The Midwife's Dictionary. By Henry Robinson, M.D. (London: The Scientific Press, Ltd. 1909. Price 1s. net.)

As this pocket dictionary is intended for midwives and obstetric or gynæcological nurses, its principal interest for the medical profession will be for those who teach or examine nurses in those sciences. From this point of view it is admirable, for while free from any pretence of being a compendium of midwifery, it gives most lucid explanations of every technical term with which midwives can reasonably be expected to become familiar. There is no doubt that a want such as this modest volume supplies has been severely felt, for it is common experience among those who examine for the C.M.B. certificates, for instance, to find candidates unaware of the meaning of many of the technical terms which they use. On one or two points (infant feeding, ophthalmia neonatorum, etc.) the author perhaps oversteps the strict limitations of a dictionary; but he has this excuse, that the fault occurs only about one or two affairs which are of most vital consequence, and on which repetition and insistence are commendable. The only real criticism that suggests itself is in regard to the phonetic spellings which are enclosed in brackets to show nurses how to pronounce the terms used; these seem to be not always constructed on a perfectly uniform plan, though the great majority are certainly satisfactory. We advise any doctor about to lecture on midwifery to nurses to recommend this dictionary to his class: not only will the latter benefit greatly, but his own labours will be much lightened.

Primer of Statistics. By W. P. and E. M. Elderton. (London: A. and C. Black, 1909. Pp. 84, 1s. 6d.)

A booklet of neat appearance, giving the elements of modern statistical methods; necessarily more baldly than in Mr. Bowley's larger manual, but with fuller reference to the developments with which the names of Sir Francis Galton (who contributes a short preface) and of Professor Pearson are associated. Revision by a literary man might have saved a small percentage of words and afforded space for more information, but this can be said of most scientific writings, and on the whole there is nothing to grumble at. A leaflet prospectus of a "Primer of Socialism" nestles among the pages. Does this indicate a "correlation"?

